# Shengjiang powder ameliorates gastric mucosal injury through activating SIRT1/FOXO1 anti-oxidative stress

**DOI:** 10.3389/fphar.2026.1855618

**Published:** 2026-08-14

**Authors:** Yihuang Liu, Xiaofeng Li, Xia Zhang, Hua Cao, Nailin Zhang, Jianhui Sun, Pingping Chen

**Affiliations:** 1 Department of Pharmacy, Hebei University of Chinese Medicine, Shijiazhuang, Hebei, China; 2 Hebei International Cooperation Center for lon Channel Function and Innovative Traditional Chinese Medicine, Hebei University of Chinese Medicine, Shijiazhuang, Hebei, China; 3 Department of Acumox and Tuina, Hebei University of Chinese Medicine, Shijiazhuang, Hebei, China; 4 Department of Chinese Medicine, Hebei University of Chinese Medicine, Shijiazhuang, Hebei, China; 5 Hebei Provincial Hospital of Chinese Medicine, Shijiazhuang, Hebei, China; 6 Hebei Key Laboratory of Turbidity Toxin Syndrome, Hebei University of Chinese Medicine, Shijiazhuang, Hebei, China

**Keywords:** chronic gastritis, forkhead box protein O1, NAD-dependent protein deacetylase sirtuin-1, oxidative stress, Shengjiang powder

## Abstract

**Introduction:**

This research sought to investigate the therapeutic efficacy and underlying mechanisms of Shengjiang Powder (SJP) in the context of chronic gastritis.

**Methods:**

A rat model of gastric mucosal injury was induced by 1-methyl-3-nitroso-1-nitrosoguanidine (MNNG) and a 10% NaCl solution, followed by treatment with either Vitacoenzyme or SJP. Histopathological changes in gastric tissues were evaluated using hematoxylin-eosin staining. Serum IL-6 levels were measured by enzyme-linked immunosorbent assay. Western blot (WB) analysis was performed to detect COX-2 expression. Immunohistochemistry was used to assess TFF1 and TFF2 expression. Serum metabolomics was conducted to identify differential metabolites and enriched pathways, while proteins related to the FOXO signaling pathway were further examined *in vivo*. WB analysis was used to detect SIRT1, FOXO1, and STAT3. CAT and SOD2 activities were measured using biochemical assays. Multiplex immunohistochemistry was used to examine SIRT1-FOXO1 co-localization and FOXO1 nuclear localization. For the *in vitro* experiments, normal human gastric epithelial cells were treated with MNNG to induce inflammatory injury, followed by SJP administration in the presence or absence of the SIRT1 inhibitor EX-527.

**Results:**

SJP alleviated gastric mucosal injury, reduced serum IL-6 levels and COX-2 expression, and significantly increased TFF1 and TFF2 expression. Metabolomics analysis identified 13 differential metabolites. The targets of these metabolites were intersected with gastritis-related targets, yielding 522 overlapping targets that were significantly enriched in the FOXO signaling pathway. *In vivo* and *in vitro* experiments showed that SJP upregulated SIRT1 and FOXO1 expression, increased CAT and SOD2 activities, inhibited STAT3 expression, and promoted FOXO1 nuclear localization. EX-527 blocked the restorative effects of SJP on FOXO1 expression and antioxidant enzyme activities.

**Conclusion:**

SJP ameliorates gastric mucosal injury and inflammation, potentially through modulation of the SIRT1/FOXO1 axis.

## Graphical Abstract

**Figure:**
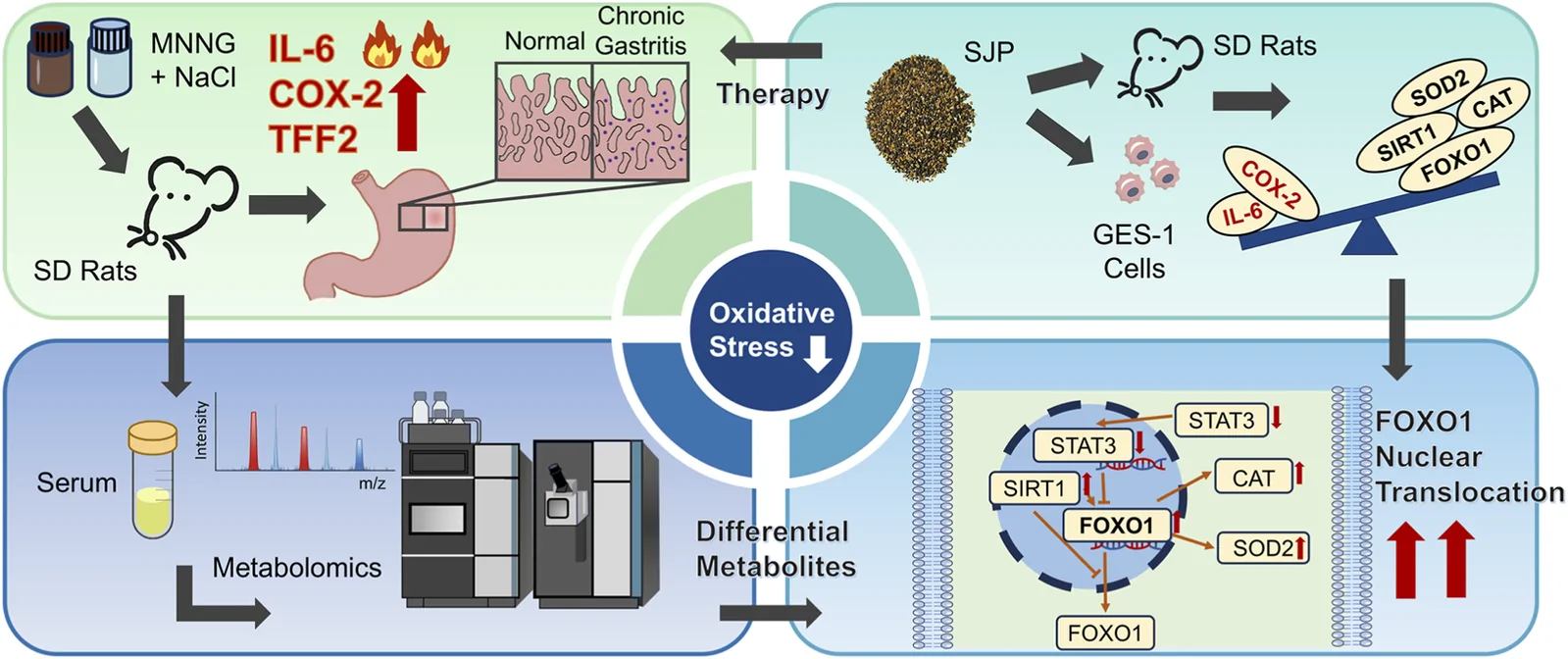

## 1 Introduction

Chronic gastritis (CG) is a common digestive disease characterized by persistent gastric mucosal injury and inflammation. It is widely accepted in the medical field that CG represents an intermediate link in the development of the disease leading to gastric cancer (GC) (Correa and Piazuelo, 2011). Therefore, effective management of CG is crucial for clinical practice, as persistent gastric inflammation may progress to atrophic changes and ultimately increase the risk of gastric cancer. However, current therapeutic strategies remain limited in fully preventing disease recurrence and progression, highlighting the need for novel and effective interventions. Thus, it is highly significant to explore alternative therapeutic strategies, particularly those derived from traditional Chinese medicine, to prevent the transition from CG to GC.

Oxidative stress is an important cause of gastric mucosal inflammation and injury. It has been shown to promote inflammation, apoptosis, and DNA damage in gastric tissue (Wang et al., 2024). Other studies have demonstrated characteristic oxidative stress alterations in chronic atrophic gastritis, including decreased levels of SOD, GSH and CAT, alongside an increase in MDA (Boeing et al., 2016; Raish et al., 2018; Chen et al., 2019; Xu B. et al., 2020). A multitude of studies have indicated that inhibiting oxidative stress protects the gastric mucosa because it reduces inflammation and inhibits the infiltration of inflammatory cells (Boeing et al., 2016; Yanaka, 2017; Raish et al., 2018). As a result, inhibiting oxidative stress may be an effective way to ameliorate gastric mucosal injury and inflammation.

Shengjiang Powder (SJP, a formula of 4 ingredients) was first recorded in Systematized Identification of *Cold Damage and Warm Pestilence* by Lishan Yang in the Qing Dynasty in 1784. This prescription is composed of *Bombyx mori Linnaeus* (Jiangcan, Chinese), *Cryptotympana pustulata Fabricius* (Chantui, Chinese), *Rheum officinale Baill*. (Dahuang, Chinese) and *Curcuma longa L*. (Jianghuang, Chinese), with a ratio of 2:1:3:4 (Yang, 2002). In modern’ clinical practice of traditional Chinese medicine, SJP is widely used as a comprehensive therapeutic prescription for various diseases, ranging from CG to GC (Sun et al., 2008; Xu H. et al., 2020; Shi et al., 2021; Guo et al., 2023). Previous studies have shown that SJP exhibits anti-inflammatory effects (Wu et al., 2025). Although SJP has been widely used in clinical practice, the molecular basis underlying the therapeutic effects of SJP in gastric mucosal injury remains poorly understood. Notably, its multi-component nature suggests that it may exert therapeutic effects through multiple molecular pathways, which remain to be elucidated. Therefore, this study investigates the protective efficacy of SJP against 1-methyl-3-nitroso-1-nitrosoguanidine (MNNG)-induced gastric mucosal injury and inflammation, with a specific focus on the SIRT1/FOXO1 axis, thereby providing novel mechanistic insights into its effects.

## 2 Methods

### 2.1 SJP preparation and medicine

Guangdong Yifang Pharmaceutical Co., Ltd. (Foshan, China) supplied the SJP formula granules, comprising *Bombyx mori* Linnaeus (Jiangcan, batch number: 1050053), *Cryptotympana pustulata* Fabricius (Chantui, batch number: 1041813), *Curcuma longa* L. (Jianghuang, batch number: 1040873), and *Rheum officinale* Baill (Dahuang, batch number: 1090813). *Bombyx mori* Linnaeus and *Cryptotympana pustulata* Fabricius used in the present study complied with local regulations regarding animal-derived medicinal materials. According to the manufacturer's specifications, 1 g of formula granules was equivalent to 3 g (*Bombyx mori* Linnaeus), 6 g (*Cryptotympana pustulata* Fabricius), 5.5 g (*Curcuma longa* L.), and 4 g (*Rheum officinale* Baill) of herbs. Based on this conversion, the adult clinical equivalent dose was approximately 3.5 g of formula granules per day.

In addition, the formula used in this study was obtained from the same batch and manufacturer as in the previous work (Liu et al., 2025), in which its major components were characterized by liquid chromatography-mass spectrometry (LC-MS). The analytical method and total ion chromatogram (TIC) for the chemical characterization of SJP are provided in Supplementary 1. The major chemical constituents of the SJP formula granules are listed in Supplementary Table S2 of Supplementary 1. Identical preparation procedures were followed to ensure compositional consistency.

Vitacoenzyme, serving as the positive control medicine in this study, was supplied by Luoyang Yilong Pharmaceutical Co., Ltd. (Luoyang, China; batch number: H41024769).

### 2.2 Animal experiments

A total of 48 male SD rats, with a weight range of 160–200 g, were procured from Beijing Vital River Laboratory Animal Technology Co., Ltd. (Beijing, China) under animal production license number: SCXK (Hebei) 2022-001, and housed in the specific pathogen-free animal room of the Experimental Animal Center of Hebei University of Chinese Medicine. This research (approval number: DWLL202203016) was approved by the Animal Ethics Committee at Hebei University of Chinese Medicine (Shijiazhuang, China).

The rats were randomly assigned to 6 groups (n = 8), designated as follows: Control, Model, Positive Control, SJP-Low, SJP-Medium, and SJP-High. To establish the gastric injury model, the remaining 40 rats were orally administered a combination solution containing 200 μg · mL^−1^ MNNG and 10% NaCl at a dose of 10 mL⋅kg^−1^ body weight, twice daily for 15 consecutive days. Concurrently, rats in the Control group were treated with an equivalent volume of normal saline (10 mL⋅kg^−1^). MNNG was purchased from Shanghai Aladdin Biochemical Technology Co., Ltd. (Cat: M105583, Shanghai, China).

The recommended daily dosage of the SJP for an adult weighing 60 kg is 7 grams, while the appropriate amount of Vitacoenzyme is 3 grams per day. The dosage of the SJP medication granules in rats is calculated to be 0.74 g per kilogram of rat body weight per day (7 g/day/60 kg × 6.3 = 0.735 g⋅kg^−1^⋅d^−1^), and Vitacoenzyme is 0.32 g per kilogram of rats’ weight per day (3 g/day/60 kg × 6.3 = 0.315 g⋅kg^−1^⋅d^−1^). Accordingly, 3 different doses of SJP were utilized: 0.19 g⋅kg^−1^⋅d^−1^ for the low-dose (SJP-Low), 0.37 g⋅kg^−1^⋅d^−1^ for the medium-dose (SJP-Medium), and 0.74 g⋅kg^−1^⋅d^−1^ for the high-dose (SJP-High). SJP in Section 2.1 was administered via gastric gavage (10 mL⋅kg^−1^) at the respective doses. Vitacoenzyme was given to the Positive Control group at 0.32 g⋅kg^−1^⋅d^−1^, and normal saline was given to the Model group. Treatments were administered continuously for 28 days. They were anesthetized with a 25% urethane (Cat: U299635, Shanghai Aladdin Biochemical Technology Co., Ltd., Shanghai, China) in normal saline at a dose of 4 mL⋅kg^−1^ prior to sample collection.

Blood was collected from the abdominal aorta. Serum was separated by centrifugation at 3,500 rpm for 15 min, then aliquoted and stored at −80 °C. Gastric tissues were collected from the greater curvature of the gastric body of each rat. Part of the tissue was fixed in 4% paraformaldehyde for paraffin embedding, while the remaining tissue was stored at −80 °C for subsequent experiments.

### 2.3 Metabolomic analysis

#### 2.3.1 Sample preparation

The serum samples were thawed at 4 °C and vortex-mixed for 1 min. An aliquot of 100 μL of serum was transferred into a 2 mL centrifuge tube, followed by the addition of 400 μL of pre-cooled methanol (−20 °C). The mixture was vortexed for 1 min to extract metabolites, and then centrifuged at 12,000 rpm for 10 min at 4 °C. All the supernatant was transferred to a new 2 mL centrifuge tube and evaporated to dryness. The residue was re-dissolved in adding 150 μL of a 2-chloro-L-phenylalanine (4 ppm) solution prepared in 80% methanol in water and stored at 4 °C. Finally, the supernatant was filtered through a 0.22 μm membrane and transferred into autosampler vials for LC-MS detection.

#### 2.3.2 Liquid chromatography analysis

The analysis was executed using a Vanquish UHPLC System (Thermo Fisher Scientific, United States). The chromatography was performed on an ACQUITY UPLC® HSS T3 column (150 × 2.1 mm, 1.8 μm) (Waters, Milford, United States). Column oven temperature: 40 °C. Automatic sampler injection volume: 2.00 µL. Flow rate: 0.25 mL⋅min^−1^. Solvent system: water (0.1% formic acid) (A) and acetonitrile (0.1% formic acid) (B). The gradient program is shown in Supplementary Table S1 of Supplementary 2.

#### 2.3.3 Mass spectrometric detection

The detection was conducted using the Orbitrap Exploris 120 (Thermo Fisher Scientific, United States) equipped with an ESI ion source. Data were acquired in Full MS followed by data-dependent MS/MS (Full MS-ddMS^2^) mode. The operational parameters were as follows: sheath gas pressure, 30 arb; auxiliary gas pressure, 10 arb. Spray voltage: 3.50 kV (Positive) and −2.50 kV (Negative). Capillary temperature: 325 °C. MS1 scanning range: 100–1000 m/z, resolution: 60,000 FWHM. MS/MS resolution: 15,000 (FWHM). Normalized collision energy: 30%. 4 data-dependent MS/MS scans per cycle. The dynamic exclusion time was set to automatic.

#### 2.3.4 Identification of chemical constituents

Raw mass spectrometry data were converted into mzXML using ProteoWizard (v3.0.8789) (Navarro-Reig et al., 2015). Peak detection, retention time alignment, and peak integration were performed using the XCMS package in R (Smith et al., 2006). Metabolite identification was achieved by matching accurate mass and MS/MS spectra against public databases, including Human Metabolome Database (HMDB) (https://www.hmdb.ca/) (Wishart et al., 2007), MassBank (https://massbank.eu/MassBank/) (Horai et al., 2010), LIPID MAPS (https://www.lipidmaps.org/) (Sud et al., 2007), mzCloud (https://www.mzcloud.org/) (Abdelrazig et al., 2020), and Kyoto Encyclopedia of Genes and Genomes (KEGG) (https://www.kegg.jp/) (Ogata et al., 1999), with a mass tolerance of 30 ppm. QC-based LOESS signal correction (Gagnebin et al., 2017) was applied to reduce systematic variation, and metabolites with a relative standard deviation (RSD) greater than 30% in QC samples were removed to ensure data reliability.

#### 2.3.5 Principal component analysis (PCA)

Unsupervised PCA of serum metabolomic data was performed using MetaboAnalyst (v. 5.0) (Pang et al., 2021). Prior to analysis, metabolite relative abundance data were log-transformed and normalized to unit variance.

#### 2.3.6 Screening of differential metabolites

Differential metabolites were screened based on the following criteria: **1.** Variable importance in projection (VIP) score **>** 1.5, obtained from the orthogonal partial least squares (OPLS) model. **2.** *P* < 0.05, determined by Student’s t-test. **3.** Fold change (FC) **>** 2 for upregulated metabolites or <0.5 for downregulated metabolites.

#### 2.3.7 Potential targets acquisition

After identifying the different metabolites, their corresponding potential targets were searched in PubChem (https://pubchem.ncbi.nlm.nih.gov/) (Kim et al., 2025), with the target species limited to *Homo sapiens*. Simultaneously, gastritis-associated targets were collected from Gene Cards (https://www.genecards.org/) (Stelzer et al., 2016) using “Gastritis” as the keyword. The intersection of targets between the identified metabolites and the disease was obtained and visualized using Draw Venn Diagram (http://bioinformatics.psb.ugent.be/webtools/Venn/).

#### 2.3.8 Gene ontology (GO)-KEGG analysis

The ClusterProfiler package in R (v. 3.18.0) (Yu et al., 2012) was utilized to perform GO-KEGG analysis on the gene set obtained from Section 2.3.7. Subsequently, the ggplot2 package (v. 3.5.0) (Wickham, 2016) was used for visualization.

#### 2.3.9 Single-gene expression difference analysis

GSE2669 was selected as core dataset from the Gene Expression Omnibus (GEO) database (http://www.ncbi.nlm.nih.gov/geo) (Edgar et al., 2002). After data preprocessing, the differential expression levels of SIRT1 and FOXO1 were analyzed using the Limma package in R (Ritchie et al., 2015), and the results were subsequently visualized with the ggplot2 package (v. 3.5.0) (Wickham, 2016).

#### 2.3.10 Protein-protein interaction (PPI) construction and analysis

Based on the results from Section 2.3.8, the proteins corresponding to the significantly enriched genes in the pathway of interest were selected to construct the PPI network. The PPI network was generated using the STRING website (v. 12.0, https://string-db.org) (Szklarczyk et al., 2023). Only interactions with a medium confidence score (score >0.4) were considered.

### 2.4 Hematoxylin-eosin (HE) staining

The gastric tissues obtained from Section 2.2 were embedded in paraffin, sectioned, and then stained with HE to examine of histopathological changes under a light microscope (Olympus BX43, Olympus Corporation, Tokyo, Japan).

### 2.5 Enzyme-linked immunosorbent assay (ELISA)

Interleukin-6 (IL-6) in serum was detected using the ELISA kit supplied by Wuhan Fine Biotech Co., Ltd. (Cat: ER0042, Wuhan, China).

### 2.6 Western blot (WB)

Radio immunoprecipitation assay (RIPA) lysate was used to extract protein samples. After determining the protein concentration, equal amounts of protein from each sample were loaded and separated via sodium dodecyl sulfate-polyacrylamide gel electrophoresis (SDS-PAGE). Then, the proteins were electrotransferred onto polyvinylidene fluoride (PVDF) membranes. The membranes were blocked and incubated with primary antibodies overnight at 4 °C. After washing, the membranes were incubated with the secondary antibodies. Finally, detection was performed using the enhanced chemiluminescence (ECL) method (GelView 6000 Plus, Guangzhou Boluteng Biotechnology Co., Ltd., Guangzhou, China). ImageJ (v. 1.52p) (Schneider et al., 2012) was used for quantitative analysis.

### 2.7 Immunohistochemistry (IHC)

The gastric tissues from Section 2.2 were paraffin embedded, sectioned, dewaxed, subjected to antigen retrieval, and treated with 3% H_2_O_2_. The sections were incubated with the primary and secondary antibody. They were developed using DAB chromogen, counterstained with hematoxylin, dehydrated in ethanol, mounted with neutral gum, and observed using a bright field microscope (Nikon Eclipse ci, Nikon Corporation, Tokyo, Japan). ImageJ (v. 1.52p) (Schneider et al., 2012) was used for quantitative analysis.

### 2.8 Antioxidant enzyme activity assay

The commercial activity assay kits of CAT (Cat: BC0205, Solarbio, Beijing, China) and SOD (Cat: A001-2-2, Nanjing Jiancheng Bioengineering Institute, Nanjing, China) were used for evaluating the activity of CAT, total SOD (T-SOD) and SOD2.

### 2.9 Cell culture and cell model construction

GES-1 (human normal gastric epithelial cell line) cells were obtained from iCell Bioscience Inc. (Cat. No. iCell-h062, Shanghai, China). The cell line was authenticated by short tandem repeat (STR) profiling and tested negative for *mycoplasma* contamination. Cells were cultured in RPMI-1640 medium supplemented with 10% fetal bovine serum (FBS), 100 IU·mL^−1^ penicillin, and 100 μg·mL^−1^ streptomycin at 37 °C in a humidified atmosphere containing 5% CO_2_.

#### 2.9.1 Determination of treatment concentrations

To evaluation of cytotoxicity, GES-1 cells in an exponential growth phase were plated into 96-well plates, with each well containing 7.5 × 10^3^ cells. SJP granules, described in Section 2.1, were dissolved in Phosphate-buffered Saline (PBS) and filtered using a 0.22 μm PES syringe filter. The cells were exposed to a range of SJP concentrations (20, 40, 80, 120, 200, 400, 600, and 800 μg⋅mL^−1^) for 24 h. Cell viability was assessed using the Cell Counting Kit-8 (CCK-8, Cat. GK10001, GlpBio Technology Inc., Montclair, USA) following the protocol provided by the manufacturer. The optical density (OD) was recorded at a wavelength of 450 nm. The percentage of cell viability was calculated using the formula: (OD_treatment/OD_control) × 100%.

To establish the cell injury model, GES-1 cells were seeded into 6-well plates at a density of 2.25 × 10^5^ cells per well and incubated with different concentrations of MNNG (10, 20, 40, 60, 80, 100 and 120 μmol⋅L^−1^) in the dark for 24 h. The concentration inducing cell damage below the IC_50_ was selected as the maximum dose for subsequent experiments. Based on this concentration, cells were treated with a gradient of MNNG (0, 2.5, 5, 10, 20, and 40 μmol⋅L^−1^) for 48 h. Protein expression levels of SIRT1 and FOXO1 were analyzed by WB (Section 2.6), and the lowest concentration that induced a significant change compared with the Control group was defined as the optimal induction concentration.

For SJP treatment, cells were co-treated with MNNG (20 μmol⋅L^−1^) and a concentration gradient of SJP (0, 10, 20, 40, and 80 μg⋅mL^−1^) for 48 h. The activities of CAT and SOD2 were measured as described in Section 2.8. The lowest concentration of SJP that significantly altered enzyme activity compared with the Model group was selected for subsequent experiments.

#### 2.9.2 SIRT1 inhibition model

Exponentially growing GES-1 cells were seeded into 6-well plates (2.25 × 10^5^ cells per well) and treated with selisistat (EX-527) (10 μmol⋅L^−1^, Cat. S1541, Selleck, Houston, USA) for 48 h to inhibit SIRT1 actinity.

#### 2.9.3 Exploration of the mechanism of SJP

Cells were subjected to the following treatments for 48 h: (1) Control (untreated); (2) MNNG (20 μmol⋅L^−1^); (3) MNNG (20 μmol⋅L^−1^) + SJP (20 μg⋅mL^−1^); and (4) MNNG (20 μmol⋅L^−1^) + SJP (20 μg⋅mL^−1^) + EX-527 (10 μmol⋅L^−1^). Subsequently, the corresponding assays were performed to evaluate protein expression and enzymatic activities as described in Sections 2.6, 2.8.

### 2.10 Multiplex immunohistochemistry (mIHC)

Paraffin sections of rat stomach tissue from Section 2.2 were sliced, then the slices were treated with the TSA kit (Cat: RS0036, ImmunoWay, Plano, USA) and primary antibody. Finally, the images were acquired randomly from each sample using a fluorescence microscope (Leica Thunder Imager DMI 8, Leica Microsystems, Wetzlar, Germany) and Leica Application Suite X (v. 3.7.5.24914). The fluorescence intensities and colocalization of FOXO1, SIRT1 and cell nuclei were analyzed using ImageJ (v. 1.52p) (Schneider et al., 2012). Plot profile analysis was performed to provide a visual representation of the colocalization pattern, and the degree of colocalization was quantitatively evaluated using the Pearson correlation coefficient (*r*).

The antibodies used for WB, IHC and mIHC are listed in Supplementary Table S2 of Supplementary 2.

### 2.11 Statistical analysis

The results are expressed as mean ± SD, with normality evaluated through the Shapiro-Wilk test. For datasets that follow a normal distribution, independent samples *t*-tests were used to compare two groups. In cases involving more than two groups, a one-way analysis of variance (ANOVA) was employed. If variances were equal, *post hoc* pairwise comparisons were conducted using Tukey’s method; however, for unequal variances, Tamhane’s T2 method was utilized for pairwise comparisons. *P* < 0.05 was deemed statistically significant for all analyses.

## 3 Results

### 3.1 SJP ameliorates gastric mucosal injury and inflammation induced by MNNG and a high concentration of NaCl

**FIGURE 1:**
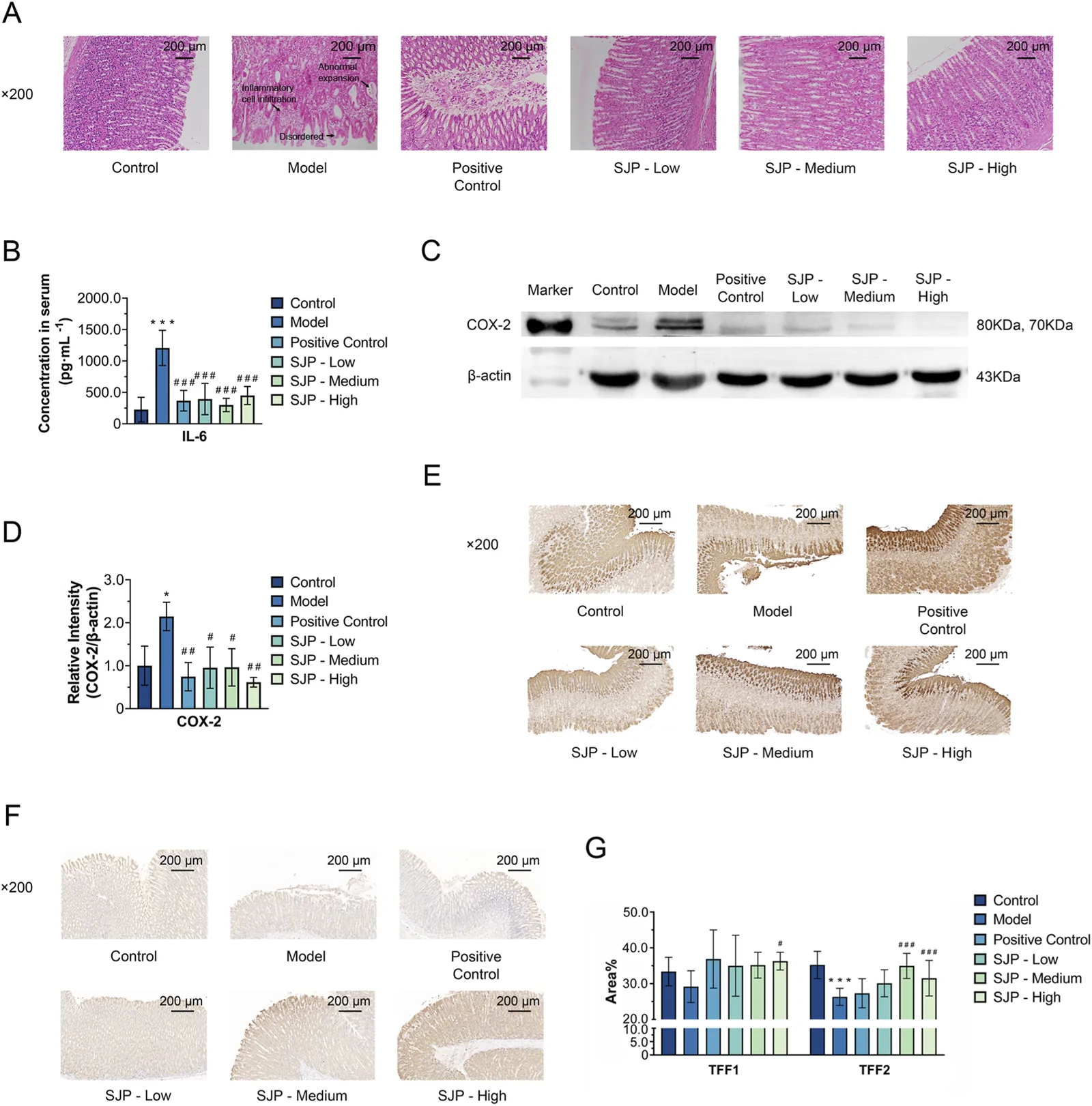
Alleviating gastric mucosal injury and inflammation with SJP. **(A)** Histopathological changes of the stomach in SD rats (HE staining, ×200). **(B)** Release of IL-6 in serum of SD rats with gastric mucosal injury regulated by SJP (*n* = 6). **(C)** and **(D)** WB analysis and quantification of COX-2 expression (*n* = 3). **(E)** and **(F)** The expression of TFF1 and TFF2 in gastric mucosa of SD rats (*n* = 6). **(G)** Quantification of TFF1 and TFF2 expression. Data are presented as mean ± SD. ^**^ *P* < 0.01 and ^***^ *P* < 0.001 vs. Control group. ^*#*^ *P* < 0.05, ^*##*^ *P* < 0.01, and ^*###*^ *P* < 0.001 vs. Model group.

In the study, gastric histomorphology indicated that the number of glands in the Model group was significantly reduced, and they were disordered, with abnormal expansion, inflammatory cell infiltration and other inflammatory manifestations as compared with the Control group. For the Positive Control group, the uniformity of the glands was restored, but the arrangement was sparse, and there was a large amount of exudate on the mucosal surface compared with the Model group. In the SJP-Low group, some epithelial tissues were still broken and shed, and the mucosa layer was thinner than that of the Control group, but compared with the Model and Positive Control group, the lamina propria cells were arranged neatly and closely. Compared with the Model group, the epithelium and lamina propria cells of the SJP-Medium and the SJP-High groups were more neatly arranged, but the gastric mucosa was still thin (Figure 1A).

Following the qualitative analysis of gastric histomorphology, a series of quantitative and semi-quantitative analyses were conducted to assess the therapeutic efficacy of SJP. The serum IL-6 levels were used to evaluate systemic inflammation, and COX-2 expression levels were used to evaluate the effect on gastric inflammation. As illustrated in Figure 1B, the results demonstrated a significant elevation of serum IL-6 levels in the Model group when compared to the Control. Conversely, there was a notable reduction in IL-6 levels observed in both the Positive Control group and all groups treated with SJP when compared to the Model. As shown in Figures 1C,D, WB analysis revealed that the Model group exhibited a significant increase in COX-2 expression levels relative to the Control, whereas a significant reduction was observed in both the Positive Control group and all SJP-treated groups when compared to the Model.

Then, the expression levels of TFF1 and TFF2 were evaluated to better characterize the observed improvement. As depicted in Figures 1E,F, TFF1 and TFF2 were detected throughout all the gastric tissues by IHC, presenting a spectrum of staining intensity ranging from yellow to brown. The percentage of positively stained cells was calculated, and the results presented in Figure 1G indicated that TFF2 level was significantly decreased when compared to the Control group. When compared to the Model group, TFF1 level was significantly elevated in the SJP-High group, while TFF2 level showed a significant increase in the SJP-Medium and -High groups.

### 3.2 Metabolomic analysis predicts that SJP ameliorates gastritis through the FOXO pathway

**FIGURE 2:**
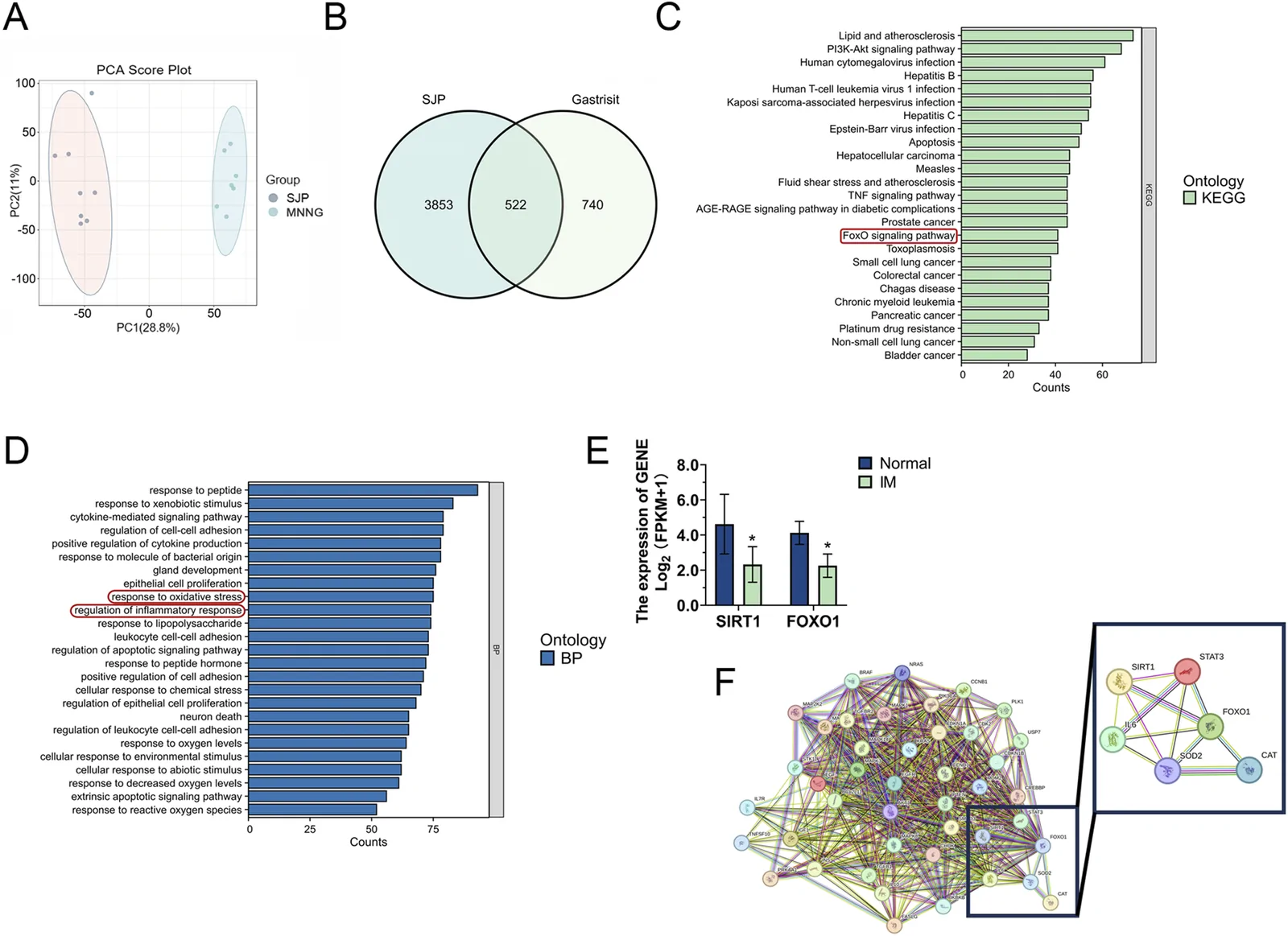
Metabolomics analysis predicted that SJP improves gastritis through the FOXO pathway. **(A)** Difference of potential metabolic pathways for the groups of the Model and SJP-High (the Model group: *n* = 8; the SJP-High group: *n* = 7). **(B)** Venn diagram showing the overlap between differentially expressed metabolites targets and disease-related targets. **(C)** Bar chart of KEGG pathway analysis. **(D)** Bar chart of BP in GO enrichment analysis. **(E)** Expression of SIRT1 and FOXO1 in normal individuals and IM patients. **(F)** The PPI network about SIRT1, FOXO1 and the intersection targets of differentially expressed metabolites targets in the FOXO pathway and disease-related targets. Data are presented as mean ± SD. ^*^ *P* < 0.05 vs. Normal group.

To explore how SJP ameliorates gastritis mechanistically, serum samples from the Model and the SJP-High group were used to analyze the metabolite profiles via principal component analysis (PCA). The results indicated that there was a significant difference between them (Figure 2A). According to the standard in Section 2.3.6, 6 upregulated metabolites and 6 downregulated metabolites were identified (Table 1). Subsequently, 4363 action targets were obtained from the 12 metabolites above. By intersecting these metabolite targets with the 1262 gastritis-associated gene targets obtained from Section 2.3.7, 522 common targets were determined (Figure 2B). Based on these common targets, a total of 3,649 enriched GO and KEGG entries were obtained (*P <* 0.05). KEGG analysis of these results showed that genes related to the FOXO signaling pathway were significantly enriched after SJP administration (Figure 2C). In the GO analysis, the Biological Process (BP) category highlighted terms such as “response to oxidative stress” and “regulation of inflammatory response” (Figure 2D), suggesting that SJP may alleviate gastric mucosal injury and inflammation by modulating oxidative stress and inflammatory pathways.

**TABLE 1:** Differentially expressed metabolites (FC > 2 and FC < 0.5).

Up or down	Compound name	Formula	M/Z	RT (min)	FC	*P*.value	VIP. value	FDR
Up	All-trans-retinoic acid	C_20_H_28_O_2_	300.1967	710.8	5.76	0.000311	1.511938	0.002197146
Up	Deoxycholic acid	C_24_H_40_O_4_	375.2859	833	7.2	0.000311	1.602249	0.002197146
Up	L-malic acid	C_4_H_6_O_5_	133.0499	94.1	3.6	0.000311	1.536344	0.002540989
Up	Phenyllactate	C_9_H_10_O_3_	165.0549	337.1	4.38	0.000622	1.509144	0.003907312
Up	Hippuric acid	C_9_H_9_NO_3_	178.0497	330	2.4	0.000311	1.538480	0.002540989
Up	Quinate	C_7_H_12_O_6_	190.9272	60	2.64	0.000311	1.645332	0.002540989
Down	2-Hydroxy-1,4-benzoquinone	C_6_H_4_O_3_	125.0237	351.2	0.02	0.000311	1.774720	0.002197146
Down	D-Ribose	C_5_H_10_O_5_	151.0345	71.4	0.03	0.000311	1.721324	0.002197146
Down	3,4-Dihydroxymandelaldehyde	C_8_H_8_O_4_	169.0495	406.4	0.13	0.000311	1.728021	0.002197146
Down	2-Oxoarginine	C_6_H_11_N_3_O_3_	173.0775	296.2	0.03	0.000311	1.775841	0.002197146
Down	Citric acid	C_6_H_8_O_7_	191.0182	68.1	0.3	0.000311	1.649850	0.002540989
Down	Guanosine	C_10_H_13_N_5_O_5_	283.2629	838.5	0.07	0.001243	1.502177	0.006394844

Gene expression data revealed that both SIRT1 and FOXO1 were significantly downregulated in patients with intestinal metaplasia (IM), a subsequent stage of CG in the disease spectrum (Correa and Piazuelo, 2011), compared with healthy individuals (Figure 2E), suggesting the potential involvement of the SIRT1/FOXO1 axis in CG progression.

The 41 genes involved in the FOXO pathway in the enrichment results were combined with FOXO1 and SIRT1 for PPI network analysis (Figure 2F), showing 567 edge connections. Additionally, the proteins in Supplementary Table S3 of Supplementary 2 were found to have meaningful correlations; therefore, they were chosen to explore the mechanism by which SJP ameliorates gastritis.

### 3.3 SJP increases FOXO1 and SIRT1, and activates CAT and SOD2

**FIGURE 3:**
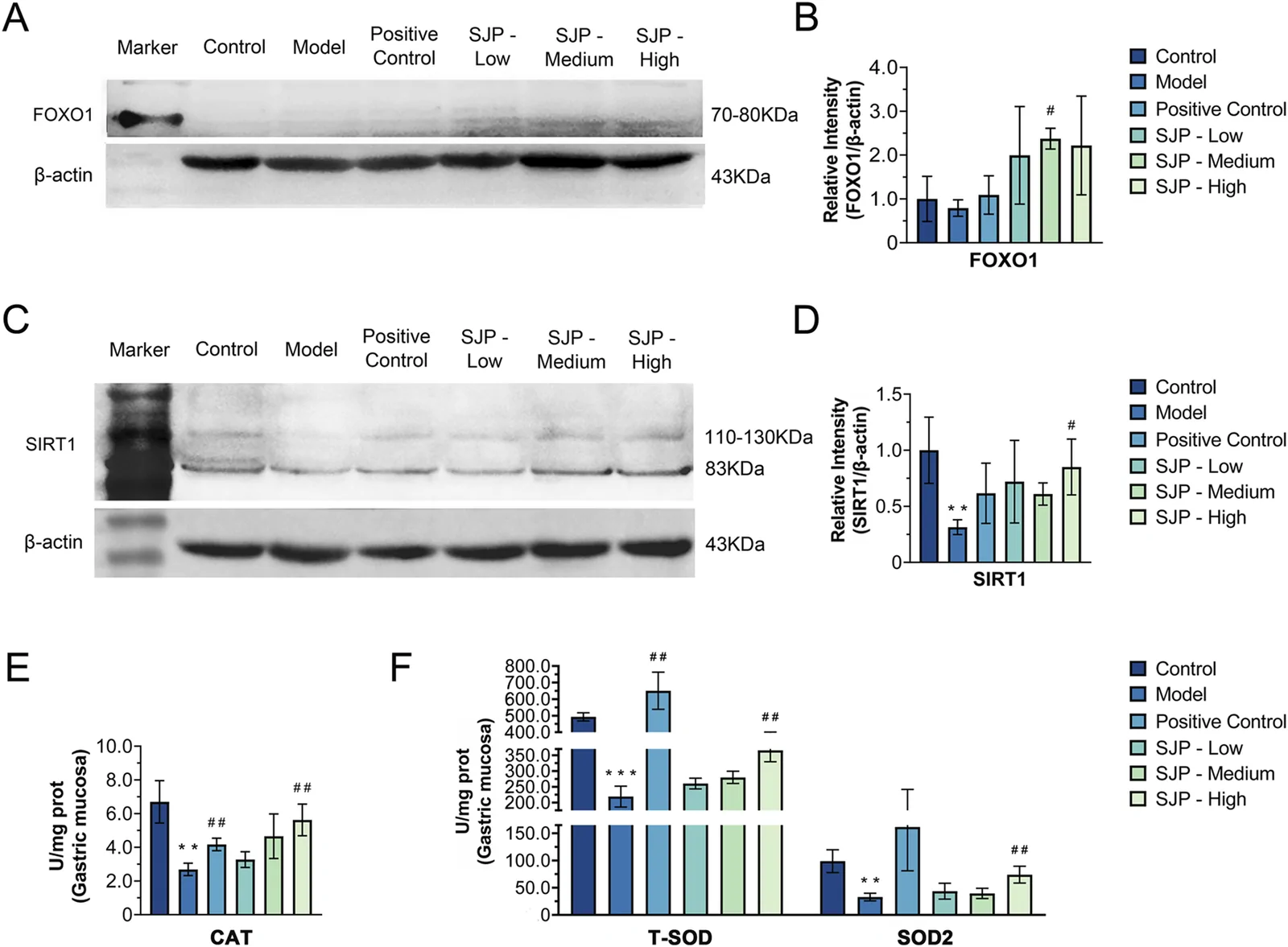
Effect of SJP on important proteins of the FOXO pathway in gastric mucosa of rats with gastric mucosal injury. **(A)** and **(B)** WB analysis and quantification of FOXO1 expression in gastric tissues of SD rats (*n* = 3). **(C)** and **(D)** WB analysis and quantification of SIRT1 expression in gastric tissues of SD rats (*n* = 3). **(E)** Activity of CAT in gastric tissues of SD rats (*n* = 6). **(F)** Activity of T-SOD and SOD2 in gastric tissues of SD rats (*n* = 6). Data are presented as mean ± SD. ^**^ *P* < 0.01 and ^***^ *P* < 0.001 vs. Control group. ^*#*^ *P* < 0.05 and ^*##*^ *P* < 0.01 vs. Model group.

WB analysis was performed to semi-quantitatively evaluate the protein expression of FOXO1 and SIRT1 in the gastric tissues of rats across different groups. The findings indicated that the FOXO1 expression levels were significantly elevated in the SJP-Medium group relative to the Model (Figures 3A,B). Moreover, SIRT1 expression levels were significantly reduced in the Model group when compared to the Control, yet an increase was observed in the SJP-High group relative to the Model (Figures 3C,D).

Furthermore, the activities of key anti-oxidative enzymes, namely, CAT, T-SOD and SOD2, were assessed. The results (Figures 3E,F) demonstrated that, compared to the Control group, the activities of CAT, T-SOD, and SOD2 were significantly lower in the Model group, whereas their activities were significantly increased in the SJP-High group when compared to the Model.

### 3.4 SJP alleviates oxidative stress-related injury in GES-1 cells, at least partly through the SIRT1/FOXO1 axis

**FIGURE 4:**
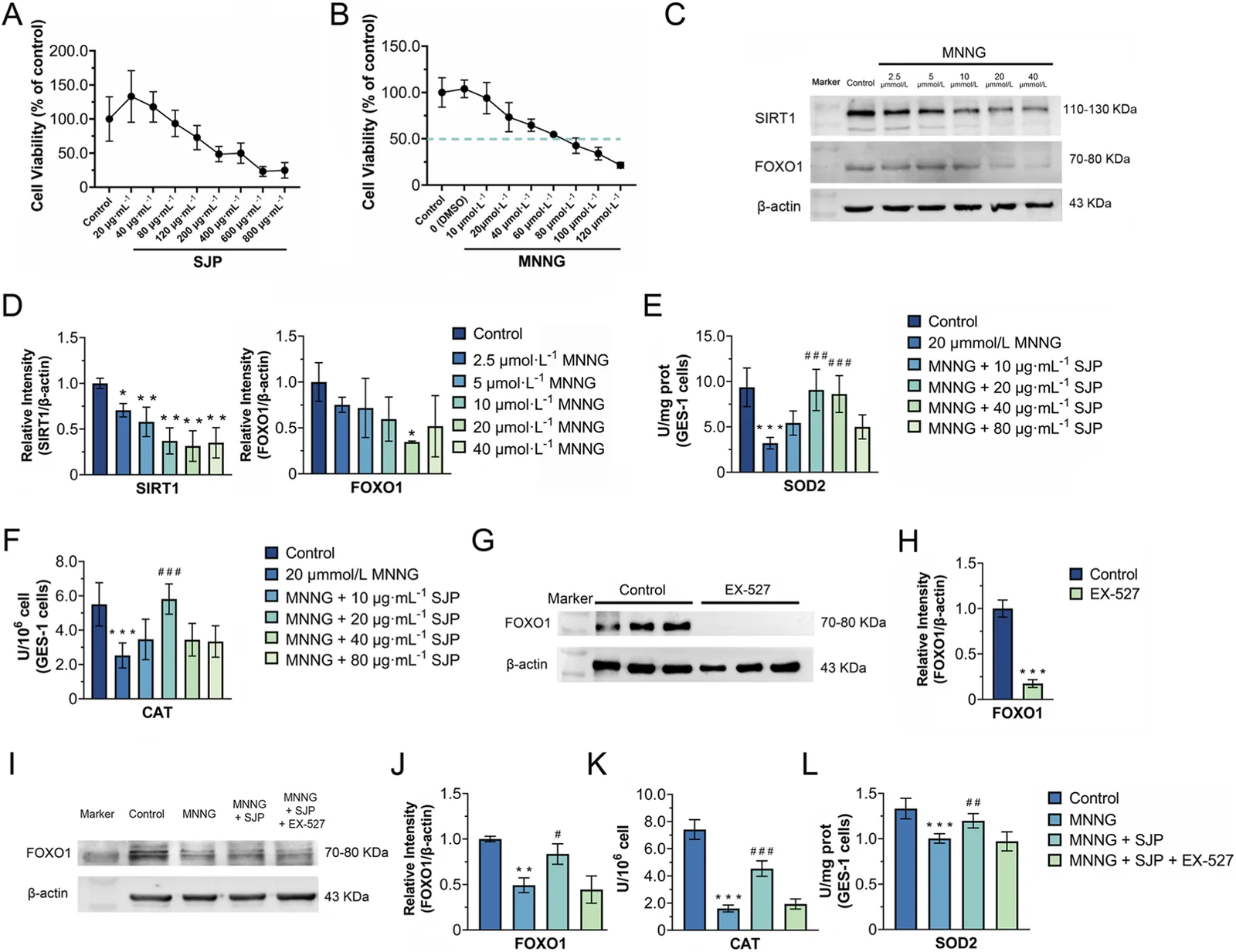
SJP alleviates oxidative stress-related injury in GES-1 cells partly through SIRT1/FOXO1. **(A)** Effects of different concentrations of SJP on the viability of GES-1 (*n* = 6). **(B)** Effects of different concentrations of MNNG on the viability of GES-1 (*n* = 6). **(C)** and **(D)** WB analysis and quantification of FOXO1 and SIRT1 expression in GES-1 cells after exposure to different concentrations of MNNG (*n* = 3). **(E)** Activity of SOD2 in GES-1 cells after exposure to 20 μmol⋅L^−1^ MNNG and different concentrations of SJP *(n* = 6). **(F)** Activity of CAT in GES-1 cells after exposure to 20 μmol⋅L^−1^ MNNG and different concentrations of SJP (*n* = 6). **(G)** and **(H)** WB analysis and quantification of FOXO1 expression following EX-527 administration (*n* = 3). **(I)** and **(J)** Expression of FOXO1 under different interventions (*n* = 3). **(K)** Activity of CAT under different interventions (*n* = 6). **(L)** Activity of SOD2 under different interventions (*n* = 6). Data are presented as mean ± SD. ^*^ *P* < 0.05, ^**^ *P* < 0.01 and ^***^ *P* < 0.001 vs. Control group. ^*##*^ *P* < 0.01 and ^*###*^ *P* < 0.001 vs. Model group.

To determine appropriate concentrations for *in vitro* experiments, the effects of SJP and MNNG on GES-1 cell viability were evaluated using the CCK-8 assay. SJP showed no cytotoxicity at concentrations ≤80 μg⋅mL^−1^ (Figure 4A). For MNNG, cell viability remained above 60% at concentrations ≤40 μmol⋅L^−1^ (Figure 4B), which was therefore considered the upper limit for subsequent *in vitro* modeling. GES-1 cells were treated with MNNG at concentrations of 2.5–40 μmol⋅L^−1^ for 48 h, and the expression levels of SIRT1 and FOXO1 were analyzed by WB. As shown in Figures 4C,D, treatment with 20 μmol⋅L^−1^ MNNG significantly decreased the expression of both SIRT1 and FOXO1 compared with the Control group. Therefore, 20 μmol⋅L^−1^ MNNG was selected to establish the cell injury model for subsequent experiments. Based on the established model (20 μmol⋅L^−1^ MNNG), GES-1 cells were further treated with SJP (10–80 μg⋅mL^−1^), and the activities of CAT and SOD2 were measured. MNNG significantly reduced CAT and SOD2 activities in GES-1 cells. Treatment with SJP (10–40 μg⋅mL^−1^) significantly restored SOD2 activity (Figure 4E), while 20 μg⋅mL^−1^ SJP significantly increased CAT activity (Figure 4F). Therefore, 20 μg⋅mL^−1^ SJP was selected for subsequent mechanistic experiments.

To examine the regulatory relationship between SIRT1 and FOXO1, GES-1 cells were treated with the SIRT1 inhibitor EX-527 (10 μmol⋅L^−1^) for 48 h. WB analysis showed that EX-527 significantly decreased FOXO1 expression (Figures 4G,H), indicating that inhibition of SIRT1 suppresses FOXO1 expression.

To further investigate whether SIRT1 mediates the regulatory effect of SJP on FOXO1, GES-1 cells were divided into 4 groups: the Control group, 20 μmol⋅L^−1^; the MNNG group, 20 μmol⋅L^−1^; MNNG +20 μg⋅mL^−1^ SJP; and 20 μmol⋅L^−1^ MNNG +20 μg⋅mL^−1^ SJP +10 μmol⋅L^−1^ EX-527. WB results showed that MNNG markedly reduced FOXO1 expression, whereas SJP significantly restored its expression. However, this restorative effect was abolished when EX-527 was co-administered (Figures 4I,J). The activities of CAT and SOD2 were further measured under the same treatment conditions. MNNG significantly decreased CAT and SOD2 activities in GES-1 cells, whereas SJP treatment restored their activities. However, this protective effect disappeared when EX-527 was co-administered (Figures 4K,L).

Taken together, these results indicate that SJP alleviates oxidative stress-related injury in GES-1 cells at least partly through regulation of the SIRT1/FOXO1 axis.

### 3.5 SJP can regulate the nuclear translocation of FOXO1

Previous studies have shown that SIRT1 can directly interact with FOXO1 and promote its nuclear localization (Hariharan et al., 2010). Therefore, the regulatory effect of SJP on the SIRT1-FOXO1 axis was further investigated.

**FIGURE 5:**
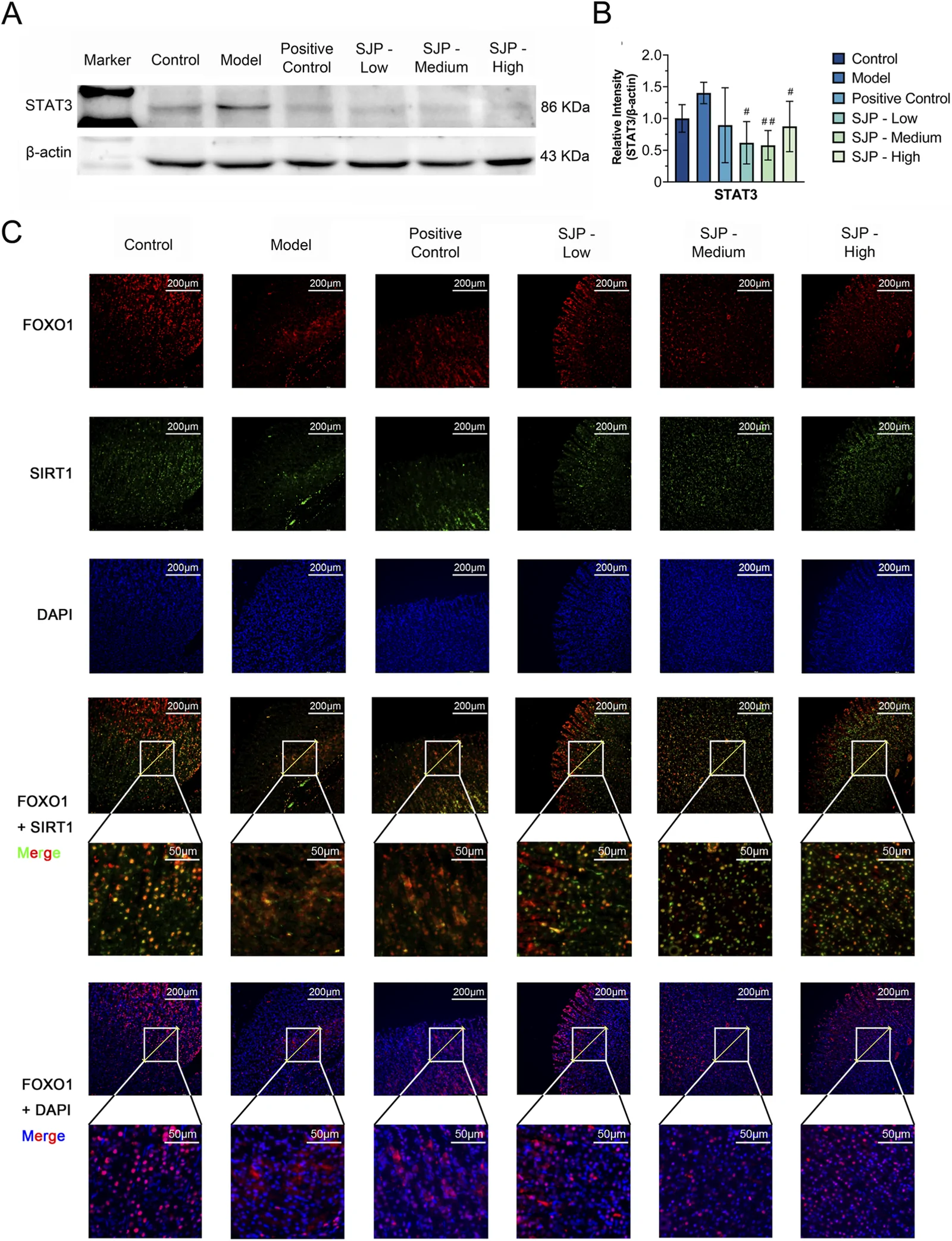
STAT3, SIRT1 and FOXO1 in gastric mucosa of rats. **(A)** and **(B)** WB assay and quantification for the expression of STAT3 (*n* = 3). **(C)** mIHC analysis of FOXO1 nuclear localization and SIRT1/FOXO1 co-expression. Data are presented as mean ± SD. ^*#*^ *P* < 0.05 and ^*##*^ *P* < 0.01 vs. Model group.

WB analysis demonstrated that the expression of STAT3 in rat gastric tissues was significantly reduced in all SJP-treated groups compared with the Model group (Figures 5A,B).

**FIGURE 6:**
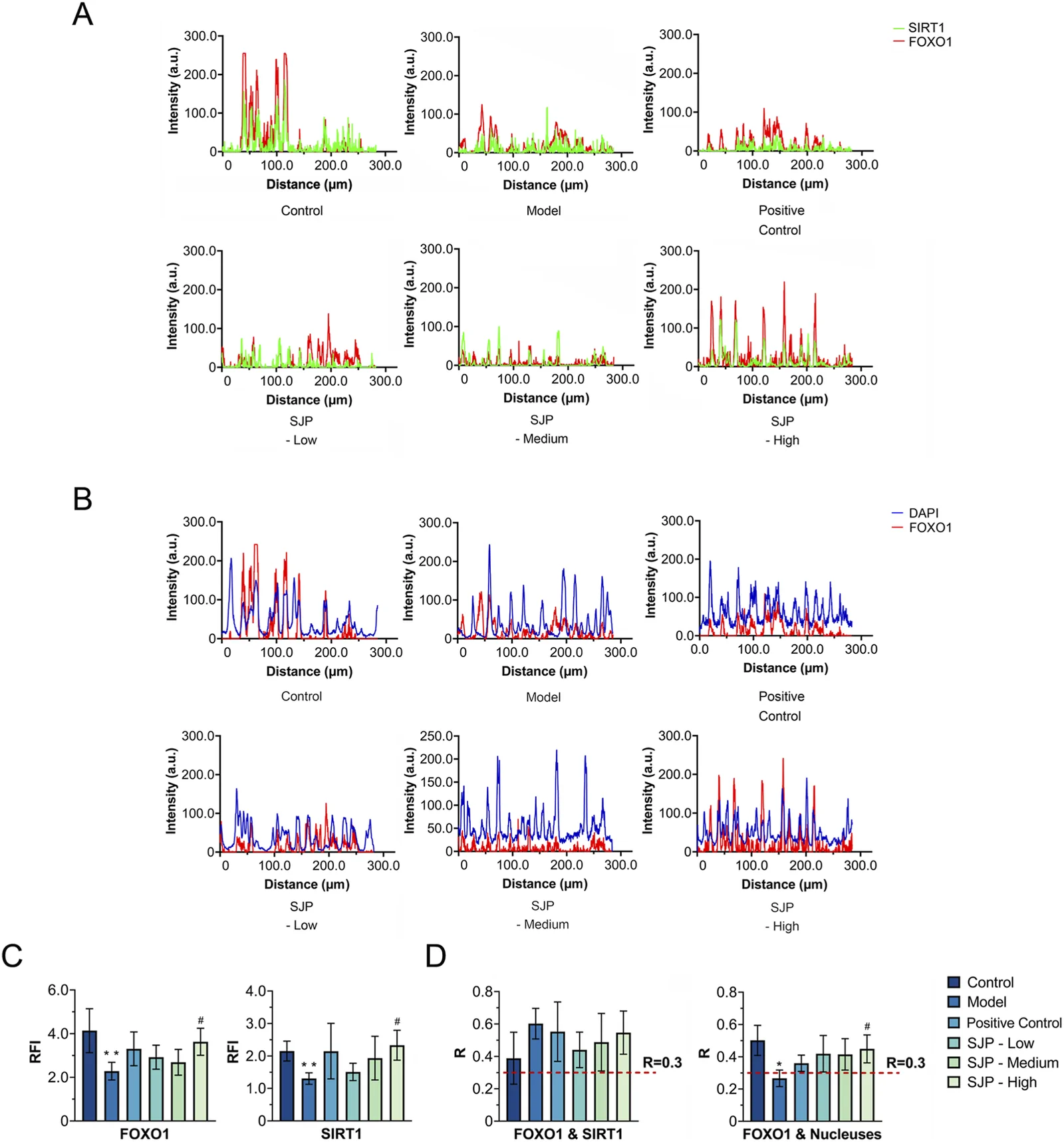
Effects of SJP on the expression and colocalization of FOXO1 and SIRT1. **(A)** Plot Profile of SIRT1 (green) and FOXO1 (red) immunofluorescence signals. **(B)** Plot Profile of nuclei (blue) and FOXO1 (red) immunofluorescence signals. **(C)** Quantitative analysis of the relative fluorescence intensity of SIRT1 and FOXO1 (*n* = 3). **(D)** Statistical analysis of *r* for the colocalization of SIRT1 with FOXO1 and FOXO1 with nuclei (*n* = 3). Data are presented as mean ± SD. ^*^ *P* < 0.05 and ^**^ *P* < 0.01 vs. Control group. ^*#*^ *P* < 0.05 vs. Model group.

Subsequently, mIHC staining was performed to visualize the cellular localization of these proteins. Compared with the Control group, the nuclear localization rate of FOXO1 decreased in the Model, and SJP significantly improved it (Figure 5C). The relative fluorescence intensities (RFI) of FOXO1 and SIRT1 were quantified. This was indicated in Figure 6C. FOXO1 levels were significantly elevated in the SJP-High group relative to the Model. Moreover, SIRT1 was significantly reduced in the Model group when compared to the Control, yet an increase was observed in the SJP-High group relative to the Model. The expression patterns of SIRT1 and FOXO1 were basically consistent with the trend of the results of the WB assay in Section 3.3.

To assess spatial localization, Plot Profiles along the diagonal in the highlighted frames in Figure 5C were obtained for FOXO1, SIRT1, and the nucleus. In parallel, co-localization was quantified using Pearson’s correlation coefficient (*r*). Figure 6A,B illustrate the spatial localization patterns of these markers. In Figure 6A, the overlapping expression profiles of SIRT1 and FOXO1 indicated that their spatial distributions were largely similar across all groups. In Figure 6B, the profiles of FOXO1 and the nucleus in the Control and the SJP-High groups were largely consistent, whereas the other groups exhibited partial differences. Subsequently, quantitative analysis using *r* confirmed these qualitative observations: in Figure 6D, *r* > 0.3 across all groups, indicating a spatial correlation between SIRT1 and FOXO1. Meanwhile, *r* was significantly reduced in the Model group compared with the Control, suggesting that gastric mucosal injury and inflammation may impair FOXO1 nuclear translocation. Conversely, *r* increased in the SJP-High group relative to the Model, indicating that SJP administration effectively restored FOXO1 nuclear localization.

## 4 Discussion

The application of SJP to gastric mucosal inflammation represents a modern reinterpretation and innovation derived from its traditional indications. Previous pharmacological studies have demonstrated that the aqueous extracts of *Bombyx mori* Linnaeus (Kim et al., 2001) and *Rheum officinale* Baill (Ma et al., 2024) possess antioxidant properties. In addition, the major bioactive constituents of *Cryptotympana pustulata* Fabricius (Xu et al., 2006; Guo et al., 2022) and *Curcuma longa* L. (Maiti et al., 2007; Samuhasaneeto et al., 2009) have been reported to exert potent anti-inflammatory activities by suppressing cytokines such as TNF-α through inhibition of the NF-κB pathway, while also exhibiting antioxidant activity. In our previous work (Liu et al., 2025), the major chemical constituents of SJP identified by LC-MS were subjected to network pharmacology analysis, which suggested that these compounds are involved in the regulation of oxidative stress and inflammatory responses.

A high concentration of NaCl can result in gastric mucosal injury (Furihata et al., 1996). MNNG is widely used to induce a series of models ranging from CG to GC in the long term (Tatematsu et al., 1975; Tong et al., 2021; Liu et al., 2024) because it results in unscheduled DNA synthesis and stimulation of replicative DNA synthesis (Furihata et al., 1984). Although epidemiological investigations show that most cases of CG are caused by *Helicobacter pylori* infection, inducing this model solely through *H. pylori* infection typically requires a prolonged period and exhibits substantial variability in both induction time and pathological alterations. As a result, the established model is often unstable. Therefore, this approach is predominantly applied to studies evaluating the antibacterial effects of drug interventions. However, as the present study focuses on the anti-inflammatory effects of SJP on the gastric mucosa and its potential to promote mucosal repair, we adopted an experimental classical modeling method involving MNNG in combination with a high-concentration NaCl solution. The body weight of rats in the Model group was reduced compared with that in the Control group, and gastric histomorphology revealed severe inflammatory manifestations in the Model group.

It was found that the alleviation of gastric mucosal injury by SJP may be achieved through the relief of inflammation. IL-6 serves as a significant pro-inflammatory cytokine that triggers a cascade of inflammatory responses via the enhancement of immune activity. COX-2 is an induced cyclooxygenase that is mainly expressed under inflammatory stimulation (Maricic et al., 1999). As shown in the results, IL-6 and COX-2 levels in the Model group were significantly increased, indicating marked systemic and local inflammatory responses. These elevations were successfully reversed in the Positive Control group, confirming the validity and reliability of the established animal model. Furthermore, administration of SJP in each treatment group also reduced IL-6 and COX-2 levels, suggesting that SJP alleviates inflammation.

Trefoil factor family proteins, including TFF1 and TFF2, play important roles in gastric physiology and pathology. They are mainly produced by gastric surface mucous cells and mucous neck or antral gland cells (Rio et al., 1988; Rasmussen et al., 1992), contributing to rapid epithelial restitution following mucosal injury (Hoffmann, 2005). In addition, TFF1 possesses anti-inflammatory properties and may participate in scavenging extracellular ROS (Heuer et al., 2020; Znalesniak et al., 2020). In the present study, SJP treatment significantly increased TFF1 expression, suggesting that restoration of the gastric mucus barrier together with anti-inflammatory activity contributes to the protective effects of SJP against gastric mucosal injury. The increase in TFF2 expression requires careful interpretation. TFF2 cooperates with MUC6 to maintain gastric mucosal integrity and contributes to the inhibition of *H. pylori* infection (Hanisch et al., 2014). However, upregulation of the TFF2/MUC6 axis has also been recognized as a feature of spasmolytic polypeptide-expressing metaplasia (SPEM) (Can et al., 2013). Nevertheless, considering the histopathological findings showing improved gastric mucosal architecture following SJP treatment, with no typical morphological features associated with SPEM progression were observed. Therefore, the upregulation of TFF2 in the present model is more likely to represent a protective or reparative response. These findings suggest that the therapeutic effects of SJP on gastric mucosal injury and inflammation are unlikely to depend on a single pathway but rather involve multiple coordinated mechanisms contributing to mucosal restoration.

As a complex traditional herbal formula containing multiple bioactive constituents, SJP may exhibit non-linear pharmacokinetic and pharmacodynamic properties, which may contribute to the observed non-linear dose-response relationship. Notably, activation of the SIRT1/FOXO1 axis was more pronounced in the SJP-Medium and SJP-High groups, which were closer to the clinically recommended dosage range, in further mechanistic studies, suggesting that while low-dose SJP is sufficient to trigger initial anti-inflammatory cascades, molecular pathway engagement may require a higher threshold.

The primary aim of the metabolomic analysis was to explore the regulatory effects of the SJP intervention on metabolic alterations under pathological conditions. Therefore, serum samples from the Model and SJP-High groups were selected for metabolomic analysis. This strategy was employed to maximize the signal-to-noise ratio and amplify SJP-induced perturbations within the endogenous metabolic network, thereby ensuring the robust capture of potential biomarkers and critical pathways while minimizing the risk of missing key targets due to weak signals at lower doses. GO-KEGG analysis suggested that SJP may exert multi-targeted therapeutic actions against oxidative stress-related gastritis. These results should reflected broader systemic metabolic shifts and provide indirect pathway-enrichment evidence, while their direct interaction with the SIRT1/FOXO1 axis remains to be fully elucidated.

Among the enriched pathways, the FOXO pathway is particularly relevant to oxidative stress regulation. In our current study, 41 targets were successfully mapped to this pivotal cascade, which plays a critical role in maintaining cellular and organismal homeostasis through regulation of stress responses (Rodriguez-Colman et al., 2024). FOXO proteins, the core components of this pathway, regulate cellular redox balance and limit damage caused by ROS (Gui and Burgering, 2022). Among the FOXO proteins, FOXO1 functions as a key transcription factor involved in oxidative stress regulation and inflammatory responses (Li et al., 2021). Activation of FOXO1 promotes transcription of antioxidant enzymes such as CAT and SOD, thereby enhancing cellular antioxidant capacity and limiting ROS-mediated damage (Xing et al., 2018). Our previous study suggested the potential involvement of the FOXO signaling pathway through network pharmacology analysis (Liu et al., 2025).

Oxidative stress is widely recognized as a major contributor to gastric mucosal injury in CG. Excessive ROS accumulation can induce inflammatory responses, apoptosis, and DNA damage in gastric tissues, thereby accelerating disease progression (Wang et al., 2024). Previous studies have reported that CG is associated with decreased levels of antioxidant enzymes such as SOD, CAT, and GSH, together with increased lipid peroxidation products such as MDA (Boeing et al., 2016; Raish et al., 2018; Chen et al., 2019; Xu B. et al., 2020). Inhibition of oxidative stress protects the gastric mucosa, attenuates inflammation, and prevents infiltration of inflammatory cells (Boeing et al., 2016; Yanaka, 2017; Raish et al., 2018). These findings indicate that restoration of antioxidant defenses represents an important strategy for protecting the gastric mucosa. SIRT1 alleviates oxidative stress by promoting FOXO1 nuclear localization and enhancing transcription of antioxidant enzymes such as CAT and SOD2 (Alcendor et al., 2007; Hsu et al., 2010). To verify the involvement of this pathway, *in vivo* experiments were performed using gastric tissues of SD rats, and *in vitro* experiments were conducted using GES-1 cells. *In vivo*, the expression levels of FOXO1 and SIRT1 were both reduced in the MNNG/NaCl-induced model; *in vitro*, exposure to 20 μmol⋅L^−1^ MNNG significantly decreased the expression levels of SIRT1 and FOXO1. These findings were consistent with previous reports in healthy individuals and patients with IM, suggesting a shared regulatory node in gastric mucosal injury progression. Under these conditions, the activities of antioxidant enzymes CAT and SOD2 were markedly reduced *in vivo* and *in vitro*, whereas treatment with SJP significantly restored both enzyme activities.

EX-527 is a highly effective and specific inhibitor of SIRT1 that suppresses its deacetylase activity. SIRT1 modulates the function of FOXO1 through post-translational modification by deacetylation (Daitoku et al., 2004; Kitamura et al., 2005; Nakae et al., 2006). Previous research has indicated that acetylation mainly influences FOXO1 function by changing its subcellular localization (Calnan and Brunet, 2008). In the current study, treatment with EX-527 at a concentration of 10 μmol⋅L^−1^ led to a significant reduction in FOXO1 protein levels, which may be linked to increased degradation of FOXO1. In cells lacking SIRT1, elevated acetylation of FOXO1 enhances its phosphorylation at Thr24 (Chae and Broxmeyer, 2011). The phosphorylated FOXO1 then interacts with 14-3-3 proteins and remains in the cytoplasm, which promotes its association with ubiquitin E3 ligases and results in degradation mediated by ubiquitination (Schäll et al., 2015). The results suggest that EX-527 effectively disrupts the SIRT1/FOXO1 pathway in the current experimental setup. To further elucidate the role of SIRT1 in this mechanism, EX-527 was employed as a pharmacological tool. SIRT1 inactivation significantly abolished the restorative effects of SJP on FOXO1 expression. Additionally, EX-527 concurrently abolished the SJP-induced recovery of CAT and SOD2 activities. These findings suggest that the antioxidant efficacy of SJP is, at least in part, dependent on the activation of the SIRT1/FOXO1 pathway.

FOXO1 must translocate to the nucleus to exert its transcriptional activity (Yan et al., 2012). Inflammatory cytokines such as IL-6 can activate the JAK/STAT3 pathway (Sansone and Bromberg, 2012), and phosphorylated STAT3 has been reported to promote FOXO1 phosphorylation (Calvier et al., 2017), resulting in cytoplasmic retention and reduced transcriptional activity (Brunet et al., 1999). In the present study, SJP treatment significantly reduced both IL-6 levels and STAT3 expression, which may facilitate FOXO1 nuclear localization and enhance its transcriptional activity.

Activation of SIRT1 has been reported to promote nuclear retention of FOXO1 and thereby enhance transcription of its target genes (Frescas et al., 2005; Hsu et al., 2010). Previous studies have also reported that H_2_O_2_-induced oxidative stress can promote FOXO1 nuclear localization (Frescas et al., 2005). However, in the present study, FOXO1 nuclear localization was significantly reduced in the Model group compared with the Control group, suggesting that complex inflammatory conditions differ from the relatively simple oxidative stress induced by H_2_O_2_ and may impair rather than activate FOXO1-mediated antioxidant responses. Because MNNG modeling decreased the expression of both SIRT1 and FOXO1, such compensatory interactions may be insufficient to maintain effective FOXO1 nuclear localization and function. After treatment with high-dose SJP, restoration of SIRT1 expression was accompanied by increased FOXO1 expression and markedly enhanced FOXO1 nuclear localization. These findings suggest that the therapeutic effects of SJP may be mediated, at least in part, through regulation of the SIRT1/FOXO1 axis by restoring SIRT1 levels and promoting FOXO1 nuclear localization, consistent with previous reports that the SIRT1-FOXO1 interaction enhances nuclear retention and stimulates antioxidant gene transcription (Hsu et al., 2010).

In the present study, a sample size of n = 6 per group was adopted for most assays, which aligns with widely accepted standards and balances statistical requirements with animal welfare principles. While this cohort size proved statistically sufficient to capture the therapeutic effects of SJP on key indicators, it may increase the risk of Type II errors. Therefore, evaluating these findings in a larger independent cohort will be a valuable direction for future studies.

In addition, the inclusion of the Control group for comparative metabolomic analysis alongside the Model and treatment groups may provide a more comprehensive framework for distinguishing disease-associated metabolic alterations from treatment-reversible metabolic changes. Future studies incorporating this strategy may further improve the understanding of metabolic remodeling during CG progression and following SJP intervention.

In summary, this study suggests that the SIRT1/FOXO1 axis is involved in the antioxidant response of SJP. Although pharmacological inhibition with EX-527 provided evidence supporting the involvement of SIRT1, this approach does not fully establish its causal or indispensable role in the observed effects of SJP. Therefore, genetic approaches, such as SIRT1-silenced or SIRT1-overexpressing cell lines, are required to further validate the specific contribution of SIRT1. Moreover, future research could focus on the identification of key bioactive components and further elucidation of the underlying molecular mechanisms, which may provide new insights into the treatment of CG and the development of novel therapeutic agents.

## 5 Conclusion

The study investigated the ameliorative effects of SJP on gastric injury to explore its therapeutic mechanisms in CG. SJP was found to alleviate gastric mucosal injury and relieve both systemic and local gastric inflammatory responses, which was partially attributed to the regulation of the SIRT1/FOXO1 axis. This modulation subsequently promoted the nuclear localization of FOXO1 and enhanced the activities of CAT and SOD2 to combat oxidative stress.

## Data Availability

Original datasets of the mass spectrometry (MS/MS) of the Serum Metabolomics of Shengjiang Powder in an MNNG-Induced Rat Model of Gastric Mucosal Injury and the Mass Spectrometry Identification Results for the Main Chemical Components of Shengjiang Powder are publicly available. This data can be found here: https://doi.org/10.6084/m9.figshare.33138410. Other original contributions presented in the study are included in the Article/Supplementary Material, further inquiries can be directed to the corresponding authors. Publicly available datasets were accessed and analyzed using the following resources: HMDB (https://www.hmdb.ca/), MassBank (https://massbank.eu/MassBank/), LIPID MAPS (https://www.lipidmaps.org/), mzCloud (https://www.mzcloud.org/), KEGG (https://www.kegg.jp/), PubChem (https://pubchem.ncbi.nlm.nih.gov/), and GeneCards (https://www.genecards.org/). Additionally, public transcriptomic data were retrieved from the GEO database (http://www.ncbi.nlm.nih.gov/geo).
