# Supplementary material for: Shengjiang powder ameliorates gastric mucosal injury through activating SIRT1/FOXO1 anti-oxidative stress
Source: Front Pharmacol. 2026 Aug 14;17:1855618. doi: 10.3389/fphar.2026.1855618 (PMC13522233; doi:10.3389/fphar.2026.1855618)
Supplement: Supplementary file 1 [file Supplementaryfile1.docx]

**Analysis of the composition of the SJP medication granules**

1. **Method**

1.1 Preparation of the SJP solution. A total of 200 mg of the SJP medication granules was weighed, and 1 mL of 80% methanol was added for samples extraction. After being mixed for 5 min and vortexed for 10 min, the mixture was centrifuged at 12,000 rpm and at 4℃ for 10 min. The resulting supernatant was carefully collected, filtered, and then analyzed.

1.2 Liquid Chromatography Analysis. The analysis was executed utilizing the UltiMate 3000 RS (Thermo Fisher Scientific, Waltham, USA). The chromatography was performed on a XB-C18 column (50 mm × 2.1 mm, 1.8 μm) (Welch, Skokie, Illinois, USA). Column oven temperature: 35℃. Automatic sampler temperature: 10.0℃. Automatic sampler injection volume: 5.00 µL. Flow rate: 0.30 mL·min^−1^. Mobile phase: (A) 0.1% formic acid in water (*v*/*v*) and (B) Methanol. The gradient program is shown in Table 1.

**Table S1 Gradient Program**

| Time (min) | A (%) | B (%) |
| --- | --- | --- |
| 0 | 98 | 2 |
| 1 | 98 | 2 |
| 5 | 80 | 20 |
| 10 | 50 | 50 |
| 15 | 20 | 80 |
| 20 | 5 | 95 |
| 25 | 5 | 95 |
| 26 | 98 | 2 |

1.3 Mass Spectrometric Detection. The detection was conducted using the Q Exactive (Thermo Fisher Scientific, USA) equipped with an ESI ion source. Scan mode: polarity switching between positive and negative ions. Detection mode: Full mass/dd-MS2. Resolution: 70,000 FWHM (Full MS) and 17,500 FWHM (dd-MS²). Scan range: 150.0–2000.0 *m*/*z*. Spray voltage (Positive): 3.80 kV. Capillary temperature: 300℃. Collision gas: High-purity argon gas (≥ 99.999%); Collision energy (N) CE: 30; Sheath gas: Nitrogen (purity ≥ 99.999%), 40 arb; Auxiliary gas (Aux gas heater temp) : nitrogen (≥ 99.999%), 350℃; Data collection time: 30.0 min.

1.4 Identification of Chemical Constituents. Data processing was performed using Compound Discoverer 2.1 software (Thermo Fisher, Waltham, USA), and the processed data were then retrieved and compared in the database (mzCloud and mzVault) to identify the specific compounds. The concentrations of the identified compounds were calculated through the correction curve equation of the component and the quantification limit. They were selected as the main active ingredient of the SJP based on the concentration level.

2. **Result**

The chromatogram of SJP is shown in Fig. 1. The major chemical constituents of the SJP formula granules are listed in Table 1. Identification of them are shown in Fig.2 to 21.

**Fig.1 Ions Total Flow Chart of Chemical Components in Shengjiang Power.**

Black: Total Ion Flow Chart in Negative Ion Mode. Red: Total Ion Flow Chart in Positive Ion Mode.

**Table S2 The Main Chemical Components of SJP.**

| **Compound Name** | **Formula** | **Retention Time (RT) (min)** | **M/Z** | **Molecular Weight** | **PPM** |
| --- | --- | --- | --- | --- | --- |
| (+)-ar-Turmerone | C_15_H_20_O | 15.945 | 217.15848 | 216.15123 | 2.533 |
| Indole-3-acrylic acid | C_11_H_9_NO_2_ | 4.425 | 188.07045 | 187.06325 | 3.475 |
| L-Phenylalanine | C_9_H_11_NO_2_ | 2.291 | 166.08625 | 165.07901 | 3.372 |
| (-)-Caryophyllene oxide | C_15_H_24_O | 16.502 | 221.18974 | 220.18255 | 2.652 |
| Curcumin | C_21_H_20_O_6_ | 12.566 | 369.13260 | 368.12545 | 1.625 |
| (+/-)9,10-dihydroxy-12Z-octadecenoic acid | C_18_H_34_O_4_ | 16.196 | 313.23831 | 314.24556 | 1.628 |
| Adenosine | C_10_H_13_N_5_O_4_ | 1.760 | 268.10373 | 267.09654 | 2.126 |
| Rubiadin | C_15_H_10_O_4_ | 12.572 | 253.05010 | 254.05749 | 1.581 |
| Betaine | C_5_H_11_NO_2_ | 0.59 | 118.08649 | 117.07923 | 4.319 |
| Glycine | C_16_H_12_O_5_ | 13.597 | 285.07541 | 284.06812 | 1.719 |
| 6-O-[(2E)-3-Phenyl-2-propenoyl]-1-O-(3,4,5-trihydroxybenzoyl)-β-D-glucopyranose | C_22_H_22_O_11_ | 10.660 | 461.10880 | 462.11612 | 1.084 |
| (15Z)-9,12,13-Trihydroxy-15-octadecenoic acid | C_18_H_34_O_5_ | 14.212 | 329.23300 | 330.24041 | 1.215 |

**Table S2 (Continued)**

| **Compound Name** | **Formula** | **Retention Time (RT) (min)** | **M/Z** | **Molecular Weight** | **PPM** |
| --- | --- | --- | --- | --- | --- |
| Ageratriol | C_15_H_24_O_3_ | 15.608 | 235.16907 | 234.16171 | 1.828 |
| L-Tyrosine | C_9_H_11_NO_3_ | 1.083 | 165.05469 | 164.0474 | 3.090 |
| Catechin | C_15_H_14_O_6_ | 7.642 | 289.07150 | 290.07879 | 1.730 |
| 2-Isopropylmalic acid | C_7_H_12_O_5_ | 5.581 | 175.06018 | 176.06747 | 2.742 |
| DL-Arginine | C_6_H_14_N_4_O_2_ | 0.52 | 175.11882 | 174.11149 | 2.741 |
| 1,6-Bis-O-(3,4,5-trihydroxybenzoyl) hexopyranose | C_20_H_20_O_14_ | 6.903 | 483.00797 | 484.08504 | 1.594 |
| Guanine | C_5_H_5_N_5_O | 2.363 | 152.05672 | 134.02292 | 0.132 |
| (+/-)12(13)-DiHOME | C_18_H_34_O_4_ | 17.088 | 295.22797 | 296.23505 | 2.269 |

Fig.2 (+)-ar-Turmerone

Fig.3 Indole-3-acrylic acid

Fig.4 L-Phenylalanine

Fig.5 (-)-Caryophyllene oxide

Fig.6 Curcumin

Fig.7 (+/-)9,10-dihydroxy-12Z-octadecenoic acid

Fig.8 Adenosine

Fig.9 Rubiadin

Fig.10 Betaine

Fig.11 Glycine

Fig.12 6-O-[(2E)-3-Phenyl-2-propenoyl]-1-O-(3,4,5-trihydroxybenzoyl)-β-D-glucopyranose

Fig.13 (15Z)-9,12,13-Trihydroxy-15-octadecenoic acid

Fig.14 Ageratriol

Fig.15 L-Tyrosine

Fig.16 Catechin

Fig.17 2-Isopropylmalic acid

Fig.18 DL-Arginine

Fig.19 1,6-Bis-O-(3,4,5-trihydroxybenzoyl) hexopyranose

Fig.20 Guanine

Fig.21 (+/-)12(13)-DiHOME
