# Supplementary material for: Shengjiang powder ameliorates gastric mucosal injury through activating SIRT1/FOXO1 anti-oxidative stress
Source: Front Pharmacol. 2026 Aug 14;17:1855618. doi: 10.3389/fphar.2026.1855618 (PMC13522233; doi:10.3389/fphar.2026.1855618)
Supplement: Supplementary file 2 [file Supplementaryfile2.docx]

**Supplementary Tables**

**Table S1** Gradient Program of Liquid chromatography analysis.

**Table S2** Antibodies used for WB, IHC and mIHC.

**Table S3** Relevance degree of the part proteins from Fig. 2F.

**Table S1 Gradient Program of Liquid chromatography analysis.**

| Time (min) | A (%) | B (%) |
| --- | --- | --- |
| 0 | 98 | 2 |
| 1 | 98 | 2 |
| 9 | 50 | 50 |
| 12 | 2 | 98 |
| 13.5 | 2 | 98 |
| 14 | 98 | 2 |
| 17 | 98 | 2 |

**Table S2 Antibodies used for WB, IHC and mIHC.**

| Method | Primary Information | Producer | | Cat. | Lot. | Host | Application |
| --- | --- | --- | --- | --- | --- | --- | --- |
| WB | β-actin | Servicebio | | GB15003-100 | AC230702004 | Rabbit | 1:2000 |
|  | COX-2 | Affinity | | #AF7003 | #3h56597 | Rabbit | 1:1000 |
|  | STAT3 | Affinity | | #AF6294 | #2i44198 | Rabbit | 1:750 |
|  | FOXO1 | | Proteintech | 18592-1-AP | 00141970 | Rabbit | 1:6000 |
|  | SIRT1 | | Selleck | F0526 | F052601 | Mouse | 1:1000 |
|  | Goat Anti-Mouse IgG HRP | | Affinity | #S0002 | #19z2345 | Goat | 1:10000 |
|  | HRP-conjugated Affinipure Goat Anti-Rabbit IgG (H+L) | | Proteintech | SA00001-2 | 20001194 | Goat | 1:10000 |
| IHC | TFF1 | | Proteintech | 13734-1-AP | 00072851 | Rabbit | 1:300 |
|  | TFF2 | | Bioss | bs-23857R | BC11136253 | Rabbit | 1:300 |
|  | Goat Anti-Rabbit IgG HRP | | Affinity | #S0001 | #56j9958 | Goat | 1:200 |
| mIHC | FOXO1 | | Proteintech | 18592-1-AP | 00141970 | Rabbit | 1:500 |
|  | SIRT1 | | Selleck | F0526 | F052601 | Mouse | 1:150 |

**Table S3 Relevance degree of the part proteins from Fig. 2F.**

| #node 1 | #node 2 | coexpression | experimentally_determined  _interaction | database_annotated | automated_textmining | combined_score |
| --- | --- | --- | --- | --- | --- | --- |
| CAT | FOXO1 | 0.062 | 0 | 0.5 | 0.13 | 0.556 |
|  | SOD2 | 0.12 | 0.252 | 0.792 | 0.37 | 0.908 |
| FOXO1 | SIRT1 | 0.109 | 0.816 | 0.9 | 0.984 | 0.999 |
|  | STAT3 | 0.075 | 0 | 0.9 | 0.644 | 0.964 |
|  | IL6 | 0.051 | 0 | 0 | 0.733 | 0.736 |
|  | SOD2 | 0.049 | 0 | 0.75 | 0.628 | 0.903 |
| IL6 | SIRT1 | 0 | 0 | 0 | 0.726 | 0.726 |
|  | STAT3 | 0.102 | 0.095 | 0.5 | 0.965 | 0.984 |
|  | SOD2 | 0.166 | 0 | 0 | 0.603 | 0.654 |
| SIRT1 | SOD2 | 0 | 0.045 | 0 | 0.772 | 0.773 |
|  | STAT3 | 0 | 0.51 | 0 | 0.642 | 0.817 |
| SOD2 | STAT3 | 0.174 | 0 | 0 | 0.441 | 0.518 |
